# (NH_4_)_2_Cd_2_Cl_3_F_3_ and (NH_4_)_2_Cd_2_Br_3_F_3_: The First Fluoride‐Containing d^10^ Metal Mixed Halides Exhibiting Superior Ultraviolet Nonlinear Optical Properties

**DOI:** 10.1002/advs.202414503

**Published:** 2025-01-28

**Authors:** Seunghun Choi, Yang Li, Yunseung Kuk, Kang Min Ok

**Affiliations:** ^1^ Department of Chemistry Sogang University 35 Baekbeom‐ro, Mapo‐gu Seoul 04107 Republic of Korea

**Keywords:** d^10^ metal systems, fluoride‐containing mixed halides, nonlinear optical materials, second‐harmonic generation, ultraviolet transparency

## Abstract

In the search for new ultraviolet (UV) nonlinear optical (NLO) materials, two novel cadmium mixed halide compounds, (NH_4_)_2_Cd_2_Cl_3_F_3_ and (NH_4_)_2_Cd_2_Br_3_F_3_, are successfully synthesized via hydrothermal methods. These compounds crystallize in the noncentrosymmetric (NCS) space group, *R*32, and are composed of distorted octahedral [CdX_3_F_3_] (X═Cl or Br) units, which extend into a 3D framework. Remarkably, both compounds demonstrate strong second‐harmonic generation (SHG) efficiencies—3.0 and 8.0 times that of KH_2_PO_4_ for the Cl‐ and Br‐containing analogs, respectively—with phase‐matching behavior observed. The SHG efficiency is attributed to the highly distorted coordination environment of the polarizable d^10^ Cd^2+^ ions, with (NH_4_)_2_Cd_2_Br_3_F_3_ benefiting further from Br's greater polarizability. Furthermore, these compounds exhibit wide bandgaps exceeding 4.2 eV, making them the first d^10^ metal mixed halide systems incorporating fluoride that are suitable for UV NLO applications. With UV absorption cut‐off edges as short as 203 nm for (NH_4_)_2_Cd_2_Cl_3_F_3_ and 243 nm for (NH_4_)_2_Cd_2_Br_3_F_3_, these materials represent a significant advancement in the development of UV‐transparent NLO materials. This study introduces a novel synthetic strategy for the design of d^10^ mixed halide systems with enhanced optical properties, offering promising candidates for future UV NLO technologies.

## Graphical abstract

Two novel fluoride‐containing d^10^ metal mixed halides, (NH_4_)_2_Cd_2_Cl_3_F_3_ and (NH_4_)_2_Cd_2_Br_3_F_3_, exhibit exceptional ultraviolet (UV) nonlinear optical (NLO) properties, including wide bandgaps and large second‐harmonic generation efficiencies. These materials demonstrate significant potential for UV applications, marking a new direction in the development of fluoride‐based NLO crystals.

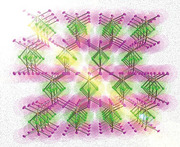

## 1 Introduction

Noncentrosymmetric (NCS) structures, which lack an inversion center, are essential for exhibiting nonlinear optical (NLO) properties. One of the most significant NLO effects is second‐harmonic generation (SHG), a process that halves the wavelength and doubles the frequency of incident light. SHG plays a critical role in various fields, including medical lasers, photolithography, and optical communication.^[^ ^1^ ^]^ For NLO materials to be effective in the ultraviolet (UV) region, they must satisfy several criteria: 1) a broad UV‐transparent range with an energy gap (*E*_g_) greater than 4.2 eV, 2) a high SHG coefficient (d*_ij_* > 0.39 pm V^−1^), and 3) sufficient birefringence to enable phase‐matching during SHG processes.^[^ ^2^ ^]^ Due to these stringent requirements, most commercially available NLO materials are based on borates and phosphates, such as β‐BaB_2_O_4_,^[^ ^3^ ^]^ LiB_3_O_5_,^[^ ^4^ ^]^ KH_2_PO_4_ (KDP),^[^ ^5^ ^]^ and KTiOPO_4_.^[^ ^6^ ^]^

To develop novel and effective NLO materials, significant research has focused on leveraging second‐order Jahn–Teller effects. This approach often utilizes d^0^ transition metal cations such as Ti^4+^, Nb^5+^, and Mo^6+^,^[^ ^7^ ^]^ as well as metals with lone electron pairs in their s orbitals, such as Pb^2+^, Bi^3+^, and Sb^3+^.^[^ ^8^ ^]^ However, a major challenge arises because asymmetric units in these systems tend to align in an antiparallel fashion, often leading to centrosymmetric crystalline products, which lack the desired NLO properties.

To enhance NLO properties and facilitate systematic synthesis, the introduction of different anions is essential. Guided by the anionic group theory, which emphasizes the role of specific functional groups, many mixed‐anion‐based NLO crystals have been successfully synthesized. For example, iodate halides such as Bi(IO_3_)F_2_^[^ ^9^ ^]^ and RbIO_2_F_2_,^[^ ^10^ ^]^ phosphate halides such as Mg_2_PO_4_Cl^[^ ^11^ ^]^ and Ba_3_P_3_O_10_Cl,^[^ ^12^ ^]^ and oxide halides such as Pb_17_O_8_Cl_18_^[^ ^1c^ ^]^ and Pb_18_O_8_Cl_15_I_5_^[^ ^13^ ^]^ illustrate the successful incorporation of various anions to create functional NLO materials. These examples highlight how different anions enhance molecular asymmetry and improve macroscopic NLO properties, underscoring their importance in material design.

In this study, a halide‐mixing strategy was used to design asymmetric units that promote the formation of NCS structures. This strategy has several advantages: 1) the coexistence of two halides with different electronegativities and sizes around the metal center introduces significant anisotropy, which disrupts inversion symmetry and encourages NCS formation; 2) highly electronegative halides lower the valence band maximum (VBM), resulting in a wider bandgap that is favorable for UV transparency; and 3) electron‐rich, polarizable halides are highly responsive to external laser fields, enhancing the NLO effect by increasing the material's nonlinear susceptibility.^[^ ^14^ ^]^ Although compounds such as Rb_2_CdCl_2_Br_2_, HgBrI, and Cs_2_HgI_2_Cl_2_ have been synthesized and studied,^[^ ^15^ ^]^ their use of heavy halides limits their ability to achieve a wide bandgap greater than 4.2 eV, making them less suitable for UV NLO applications. To address this, we designed two new NCS materials, (NH_4_)_2_Cd_2_Cl_3_F_3_ and (NH_4_)_2_Cd_2_Br_3_F_3_, based on the following principles: 1) the combination of fluoride with other halides produces a larger size disparity, which increases anisotropy around the metal center; 2) fluoride's high electronegativity effectively lowers the VBM, leading to a wider bandgap;^[^ ^16^ ^]^ 3) cadmium halide adducts tend to form 6‐coordinate octahedral environments, which are conducive to out‐of‐center distortion;^[^ ^17^ ^]^ and 4) the high polarizability of the d^10^ Cd^2+^ cation, in combination with electron‐rich halides, enhances NLO efficiency.^[^ ^18^ ^]^

Although several mixed halide compounds have been reported, most research has focused on heavy halides, which limit UV transparency due to narrower bandgaps. In contrast, our design incorporates light and highly electronegative fluoride to achieve both enhanced NLO properties and a wide UV‐transparent bandgap. To the best of our knowledge, these are the first d^10^ metal mixed halide NLO materials containing fluoride, with a bandgap exceeding 4.2 eV, making them strong candidates for UV NLO applications.

Herein, we present the synthesis, crystal structure, and NLO properties of two new mixed‐halide compounds, (NH_4_)_2_Cd_2_Cl_3_F_3_ and (NH_4_)_2_Cd_2_Br_3_F_3_. The SHG efficiencies of (NH_4_)_2_Cd_2_Cl_3_F_3_ and (NH_4_)_2_Cd_2_Br_3_F_3_ were found to be 3.0 and 8.0 times higher than that of KDP, respectively. These compounds also exhibit a broader UV transparency range and enhanced NLO performance compared to many reported UV NLO crystals, owing to the incorporation of fluoride. This study introduces a new approach to synthesizing UV NLO materials using mixed halides, underscoring their potential as promising candidates for advanced UV NLO applications.

## 2 Results and Discussion

**Figure 1:**
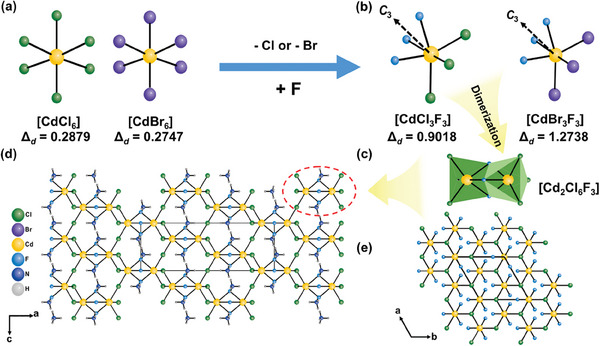
a) Symmetric [CdCl_6_] and [CdBr_6_] units in NH_4_CdCl_3_ and NH_4_CdBr_3_. b) C_3_‐distorted [CdCl_3_F_3_] and [CdBr_3_F_3_] octahedra formed by replacing Cl or Br with F in (NH_4_)_2_Cd_2_Cl_3_F_3_ and (NH_4_)_2_Cd_2_Br_3_F_3_. c) A [Cd_2_Cl_6_F_3_] dimer created through face‐sharing F^−^ anions. The complete 3D framework of (NH_4_)_2_Cd_2_Cl_3_F_3_ viewed in the d) *ac*‐ and e) *ab*‐planes.

The single crystal X‐ray diffraction analysis revealed that the isostructural title compounds crystallize in the NCS space group, *R*32 (no. 155), belonging to the trigonal crystal system (Table S1, Supporting Information).^[^ ^19^ ^]^ Since both compounds share the same crystal structure, the structure of (NH_4_)_2_Cd_2_Cl_3_F_3_ will be used to describe them. The asymmetric unit consists of one Cd^2+^ cation, three F^−^ anions, and three additional Cl^−^ anions, forming an octahedral [CdCl_3_F_3_] unit. In previously reported structures of NH_4_CdCl_3_ and NH_4_CdBr_3_, the [CdCl_6_] and [CdBr_6_] octahedra exhibit nearly identical Cd─Cl and Cd–Br bond lengths in the trans positions, with bond angles close to 180° (**Figure** **1**a), resulting in minimal out‐of‐center distortion. However, in the title compounds, different halide ions are located in the transpositions. In (NH_4_)_2_Cd_2_Cl_3_F_3_, the trans Cd─F bond length is 2.294(3) Å, the Cd─Cl bond length is 2.5725(6) Å, and the F─Cd─Cl bond angle is 161.37(7)°. In (NH_4_)_2_Cd_2_Br_3_F_3_, the trans Cd─F bond length is 2.298(3) Å, the Cd─Br bond length is 2.6882(4) Å, and the F─Cd─Br bond angle is 163.20(9)°, indicating significant distortion. The out‐of‐center distortion (Δ_d_) for the constituting octahedra was quantified using a previously reported equation.^[^ ^20^ ^]^ Using this method, the distortion magnitudes for [CdCl_3_F_3_] in (NH_4_)_2_Cd_2_Cl_3_F_3_ and [CdBr_3_F_3_] in (NH_4_)_2_Cd_2_Br_3_F_3_ were calculated to be 0.90 and 1.28, respectively, indicating substantial distortion since both values exceed 0.80.^[^ ^21^ ^]^ In other words, in both (NH_4_)_2_Cd_2_Cl_3_F_3_ and its isostructural analog (NH_4_)_2_Cd_2_Br_3_F_3_, the [CdX_3_F_3_] octahedra (X═Cl^−^ or Br^−^) exhibits a highly distorted coordination environment. It should be noted that the octahedral distortion magnitudes for the single‐halide compounds, NH_4_CdCl_3_ and NH_4_CdBr_3_, were calculated to be 0.29 and 0.27, respectively, both of which are below 0.5, corresponding to “no distortion.” In (NH_4_)_2_Cd_2_Cl_3_F_3_, three F^−^ anions occupy one face of the octahedron, while three Cl^−^ anions occupy the opposite face, causing significant distortion along the *C*_3_ axis (Figure 1b). The F^−^ anions of one unit form face‐sharing interactions with adjacent F^−^ anions, creating a [Cd_2_Cl_6_F_3_] dimer (Figure 1c). The six terminal Cl^−^ anions connect these dimers via corner‐sharing, forming a 3D [Cd_2_Cl_6_F_6_] anionic framework (Figure 1d,e). The NH_4_^+^ cations are located between the anionic framework, balancing the overall charge. Bond valence sum (BVS) calculations gave values of 2.04 for Cd^2+^, 0.80 for Cl^−^, and 0.54 for F^−^. The underestimated BVS value for F^−^ suggests strong hydrogen bonding between the N─H groups of NH_4_^+^ and the F^−^ anions.

**Figure 2:**
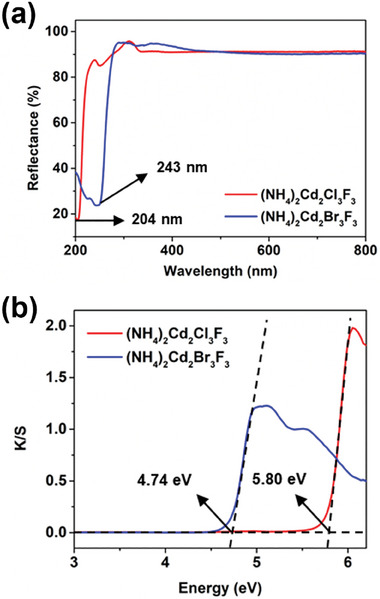
a) UV–vis diffuse reflectance spectra and b) experimental bandgaps derived from the UV–vis spectra using the Kubelka–Munk function for (NH_4_)_2_Cd_2_Cl_3_F_3_ and (NH_4_)_2_Cd_2_Br_3_F_3_.

The UV–vis diffuse reflectance spectra revealed that (NH_4_)_2_Cd_2_Cl_3_F_3_ and (NH_4_)_2_Cd_2_Br_3_F_3_ exhibit short UV absorption cut‐off edges at 203 and 243 nm, respectively (**Figure** **2**a). These values fall within the solar‐blind UV region (200−280 nm), indicating optical stability. Using the Kubelka–Munk function, the corresponding bandgap energies were calculated to be 5.80 and 4.74 eV, respectively (Figure 2b). The ability of these compounds to maintain transparency over such a wide wavelength range is attributed to the presence of highly electronegative fluorides in their composition. These properties suggest that the title compounds are promising candidates for short‐wave UV applications.

Thermogravimetric analysis (TGA) was conducted to evaluate the thermal stability of the title compounds. The TGA data indicated that both compounds remained stable up to 200 °C, after which significant weight loss was observed. Powder X‐ray diffraction (PXRD) analysis of the residue confirmed the formation of CdF_2_ and CdCl_2_ for (NH_4_)_2_Cd_2_Cl_3_F_3_, and CdBr_2_ for (NH_4_)_2_Cd_2_Br_3_F_3_. This weight loss was attributed to the decomposition of NH₄⁺ cations, likely volatilizing as NH_3_, HF, and HX (X═Cl for (NH_4_)_2_Cd_2_Cl_3_F_3_ and Br for (NH_4_)_2_Cd_2_Br_3_F_3_) above 200 °C. The resulting residues remained stable until ≈700 °C, at which point a second weight loss was observed. PXRD analysis at this stage revealed that the remaining material was CdO, suggesting that the cadmium halides had reacted with atmospheric oxygen (Figures S4 and S6, Supporting Information). The final mass was notably low, likely due to the sublimation of cadmium halides at elevated temperatures.

The electronic structures and bandgaps of the title compounds were investigated using the density functional theory (DFT) calculations (Figures S13 and S14, Supporting Information). The calculations showed direct bandgaps of 4.31 eV for (NH_4_)_2_Cd_2_Cl_3_F_3_ and 3.77 eV for (NH_4_)_2_Cd_2_Br_3_F_3_, corresponding to UV cut‐off edges at 287.7 and 328.9 nm, respectively. The calculated bandgaps are slightly smaller than the experimental values, which can be attributed to the inherent limitations of the exchange‐correlation function used in DFT calculations.^[^ ^22^ ^]^ The electronic structures of both compounds were further analyzed through the partial density of states (PDOS). For (NH_4_)_2_Cd_2_Cl_3_F_3_, the PDOS analysis revealed that the VBM primarily originates from F 2p and Cl 3p orbitals, while the conduction band minimum is mainly derived from Cd 4s orbitals (Figure S15a, Supporting Information). A similar trend was observed for (NH_4_)_2_Cd_2_Br_3_F_3_, except that the Br 4p orbital predominantly contributes to the VBM (Figure S15b, Supporting Information). The optical properties of the material are primarily determined by these frontier orbitals, indicating that they arise from the distorted [CdX_3_F_3_] units.^[^ ^23^ ^]^

**Figure 3:**
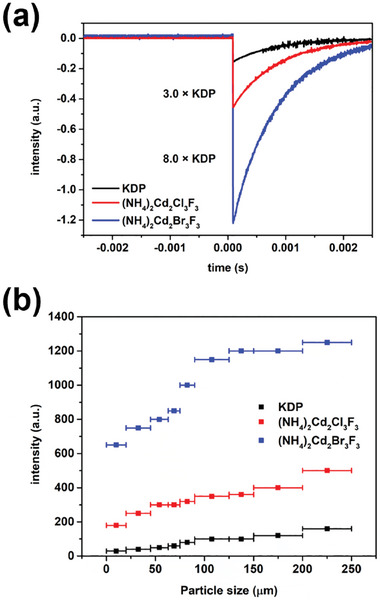
a) Oscilloscope traces of samples with particle sizes of 200–250 µm, and b) plots of SHG response versus particle size for KDP, (NH_4_)_2_Cd_2_Cl_3_F_3_, and (NH_4_)_2_Cd_2_Br_3_F_3_.

**Figure 4:**
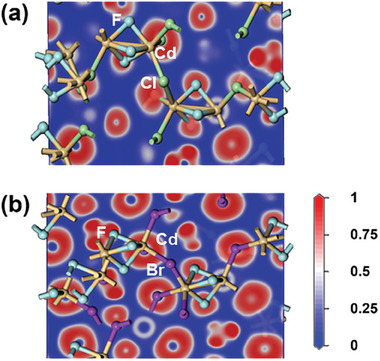
ELF contour maps for a) (NH_4_)_2_Cd_2_Cl_3_F_3_ and b) (NH_4_)_2_Cd_2_Br_3_F_3_ (light brown, Cd; cyan, F; green, Cl; purple, Br).

Since both (NH_4_)_2_Cd_2_Cl_3_F_3_ and (NH_4_)_2_Cd_2_Br_3_F_3_ crystallize in the NCS space group, *R*32, SHG measurements were conducted using the modified Kurtz–Perry method with a 1064 nm light source. SHG responses were recorded for different particle sizes of both compounds, revealing that (NH_4_)_2_Cd_2_Cl_3_F_3_ and (NH_4_)_2_Cd_2_Br_3_F_3_ exhibited large SHG efficiencies of ≈3.0 and 8.0 times that of KDP, respectively (**Figure** **3**a). Furthermore, the SHG efficiency increased with particle size, indicating phase‐matching behavior (Figure 3b). Although DFT and local dipole moment calculations showed significant distortions in the [CdX_3_F_3_] units of both compounds, their contribution to the SHG intensity is likely limited due to the fact that they crystallize in the nonpolar space group, *R*32. However, the presence of highly polarizable d^10^ cations and halides, as seen in the 2D electron localization function (ELF) contour maps (**Figure** **4**), plays a critical role in the strong SHG response. The difference in SHG efficiency between (NH_4_)_2_Cd_2_Cl_3_F_3_ and (NH_4_)_2_Cd_2_Br_3_F_3_ can be attributed to both the greater out‐of‐center distortion in the latter and the higher polarizability of Br^−^ compared to Cl^−^. The ELF contour maps show that in (NH_4_)_2_Cd_2_Cl_3_F_3_, the localized electron density around F and Cl indicates moderate polarizability, while in (NH_4_)_2_Cd_2_Br_3_F_3_, the more extended electron distribution around Br^−^ reflects much higher polarizability. This enhanced electron delocalization around Br contributes to a stronger NLO response, making (NH_4_)_2_Cd_2_Br_3_F_3_ more sensitive to external laser fields. Consequently, (NH_4_)_2_Cd_2_Br_3_F_3_ demonstrates superior SHG performance compared to (NH_4_)_2_Cd_2_Cl_3_F_3_.^[^ ^24^ ^]^

Birefringence (*Δn*) measurements for transparent crystals of both (NH_4_)_2_Cd_2_Cl_3_F_3_ and (NH_4_)_2_Cd_2_Br_3_F_3_ were performed using a polarized microscope (ZEISS Axiolab 5) equipped with a 546.1 nm monochromator. For (NH_4_)_2_Cd_2_Cl_3_F_3_, the retardation was measured at 3619.96 nm, with a crystal thickness of 41.413 µm, resulting in a calculated birefringence of 0.08741. For (NH_4_)_2_Cd_2_Br_3_F_3_, the retardation was found to be 1992.90 nm, and the crystal thickness was measured at 17.926 µm, yielding a birefringence value of 0.1112. Both compounds exhibit moderate birefringence, which is sufficient to explain the phase‐matching behavior in SHG.

**Figure 5:**
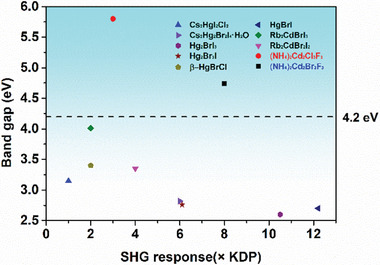
NLO properties and bandgaps of d^10^ metal mixed halide compounds.

**Figure** **5** compares the title compounds with previously reported d^10^ metal mixed halide systems. While these previously reported materials also display high anisotropy due to the presence of different halides, leading to strong SHG efficiencies that surpass those of KDP, most research has focused on their applications in the visible and infrared (IR) regions. Notably, no materials with bandgaps wider than 4.2 eV−necessary for UV NLO applications have been reported in these systems. This gap in UV‐capable materials is largely attributed to the presence of fluoride, as fluoride anions were not considered suitable for IR applications. In contrast, the compounds introduced in this study, (NH_4_)_2_Cd_2_Cl_3_F_3_ and (NH_4_)_2_Cd_2_Br_3_F_3_, are the first examples of d^10^ metal mixed halides incorporating F^−^. The high electronegativity of F significantly lowers the VBM, enabling the formation of a wide bandgap exceeding 4.2 eV. This breakthrough offers a new pathway for the development of UV NLO materials.

## 3 Conclusion

In summary, crystals of (NH_4_)_2_Cd_2_Cl_3_F_3_ and (NH_4_)_2_Cd_2_Br_3_F_3_ were successfully synthesized via hydrothermal methods. Both compounds crystallized in the NCS space group, *R*32, and exhibited isostructural relationships. They consist of highly distorted octahedral units, [CdX_3_F_3_], which form dimers, [Cd_2_X_6_F_3_], and assemble into a 3D framework, [Cd_2_X_3_F_3_]_∞_. Notably, these compounds demonstrated strong SHG efficiencies—3.0 and 8.0 times higher than that of KDP for (NH_4_)_2_Cd_2_Cl_3_F_3_ and (NH_4_)_2_Cd_2_Br_3_F_3_, respectively—along with phase‐matching behavior. The high SHG efficiency was attributed to the strongly distorted coordination environment of the highly polarizable d^10^ Cd^2+^ ions, with (NH_4_)_2_Cd_2_Br_3_F_3_ benefiting further from the greater polarizability of Br. Additionally, both compounds exhibited short UV cut‐off edges, marking the first example of F^−^ incorporation into d^10^ metal mixed halide systems. This work highlights these materials as promising candidates for UV NLO applications and introduces a novel synthetic strategy for such compounds.

## Conflict of Interest

The authors declare no conflict of interest.

## Supporting information



Supporting Information

## Data Availability

The data that support the findings of this study are available from the corresponding author upon reasonable request.

## References

[1] a) K. M. Ok , E. O. Chi , P. S. Halasyamani , Chem. Soc. Rev. 2006, 35, 710; 16862271 10.1039/b511119f

[2] a) P. F. Li , C. L. Hu , F. Kong , J.‐G. Mao , Angew. Chem., Int. Ed. 2023, 62, e202301420; 10.1002/anie.202301420 36847469

[3] C. Chen , B. Wu , A. Jiang , G. You , Sci. Sin. Ser. B. 1985, 28, 235.

[4] C. Chen , Y. Wu , A. Jiang , B. Wu , G. You , R. Li , S. Lin , J. Opt. Soc. Am. B. 1989, 6, 616.

[5] G. Samara , Ferroelectrics 1973, 5, 25.

[6] J. D. Bierlein , H. Vanherzeele , J. Opt. Soc. Am. B. 1989, 6, 622.

[7] a) B.‐W. Liu , X.‐M. Jiang , H.‐Y. Zeng , G.‐C. Guo , J. Am. Chem. Soc. 2020, 142, 10641; 32469217 10.1021/jacs.0c04738

[8] a) M.‐L. Liang , C.‐L. Hu , F. Kong , J.‐G. Mao , J. Am. Chem. Soc. 2016, 138, 9433; 27428359 10.1021/jacs.6b06680

[9] F. F. Mao , C. L. Hu , X. Xu , D. Yan , B. P. Yang , J. G. Mao , Angew. Chem. 2017, 129, 2183.

[10] Q. Wu , H. Liu , F. Jiang , L. Kang , L. Yang , Z. Lin , Z. Hu , X. Chen , X. Meng , J. Qin , Chem. Mater. 2016, 28, 1413.

[11] J. X. Zhang , Q. G. Yue , S. H. Zhou , X. T. Wu , H. Lin , Q. L. Zhu , Angew. Chem., Int. Ed. 2024, 63, e202413276. 10.1002/anie.202413276 39132935

[12] P. Yu , L.‐M. Wu , L.‐J. Zhou , L. Chen , J. Am. Chem. Soc. 2014, 136, 480. 24354457 10.1021/ja411272y

[13] X. Chen , Q. Jing , K. M. Ok , Angew. Chem., Int. Ed. 2020, 59, 20323. 10.1002/anie.202009541 32851746

[14] M. Liang , Y. Zhang , E. Izvarin , M. J. Waters , J. M. Rondinelli , P. S. Halasyamani , Chem. Mater. 2024, 36, 2113.

[15] a) Y. Dang , X. Meng , K. Jiang , C. Zhong , X. Chen , J. Qin , Dalton Trans. 2013, 42, 9893; 23695292 10.1039/c3dt50291k

[16] a) J. Chen , C.‐L. Hu , J.‐G. Mao , Sci. China Mater 2021, 64, 400;

[17] a) M. Borsari , Encycl. Inorg. Bioinorg. Chem. 2011, 1;

[18] a) Y.‐C. Hao , X. Xu , F. Kong , J.‐L. Song , J.‐G. Mao , CrystEngComm 2014, 16, 7689;

[19] Deposition Numbers 2393782 (for (NH4)2Cd2Cl3F3) and 2393783 (for (NH4)2Cd2Br3F3) Contain the Supplementary Crystallographic Data for This Paper. These Data are Provided Free of Charge by the Joint Cambridge Crystallographic Data Centre and Fachinformationszentrum Karlsruhe Access Structures Service.

[20] P. S. Halasyamani , Chem. Mater. 2004, 16, 3586.

[21] Z. Bai , K. M. Ok , Coord. Chem. Rev. 2023, 490, 215212.

[22] a) H. Mizoguchi , P. M. Woodward , Chem. Mater. 2004, 16, 5233;

[23] D. Avci , A. l. Başoğlu , Y. Atalay , Int. J. Quantum Chem. 2011, 111, 130.

[24] a) G. Zou , C. Lin , H. Jo , G. Nam , T. S. You , K. M. Ok , Angew. Chem., Int. Ed. 2016, 128, 12257; 10.1002/anie.201606782 27555114

