# Supplementary material for: (NH4)2Cd2Cl3F3 and (NH4)2Cd2Br3F3: The First Fluoride‐Containing d10 Metal Mixed Halides Exhibiting Superior Ultraviolet Nonlinear Optical Properties
Source: Adv Sci (Weinh). 2025 Jan 28;12(11):2414503. doi: 10.1002/advs.202414503 (PMC11923861; doi:10.1002/advs.202414503)
Supplement: Supplementary file 1 — Supporting Information [file ADVS-12-2414503-s001.docx]

Supporting Information
©Wiley-VCH 2021
69451 Weinheim, Germany

(NH_4_)_2_Cd_2_Cl_3_F_3_ and (NH_4_)_2_Cd_2_Br_3_F_3_: The First Fluoride-Containing d^10^ Metal Mixed Halides Exhibiting Superior Ultraviolet Nonlinear Optical Properties

Seunghun Choi, Yang Li, Yunseung Kuk, and Kang Min Ok*

**Abstract:** In the search for new ultraviolet (UV) nonlinear optical (NLO) materials, two novel cadmium mixed halide compounds, (NH_4_)_2_Cd_2_Cl_3_F_3_ and (NH_4_)_2_Cd_2_Br_3_F_3_, have been successfully synthesized via hydrothermal methods. These compounds crystallize in the noncentrosymmetric (NCS) space group, *R*32 and are composed of distorted octahedral [CdX_3_F_3_] (X = Cl or Br) units, which extend into a 3D framework. Remarkably, both compounds demonstrate strong second-harmonic generation (SHG) efficiencies—3.0 and 8.0 times that of KH_2_PO_4_ for the Cl- and Br-containing analogs, respectively—with phase-matching behavior observed. The SHG efficiency is attributed to the highly distorted coordination environment of the polarizable d^10^ Cd^2+^ ions, with (NH_4_)_2_Cd_2_Br_3_F_3_ benefiting further from Br's greater polarizability. Furthermore, these compounds exhibit wide band gaps exceeding 4.2 eV, making them the first d^10^ metal mixed halide systems incorporating fluoride that are suitable for UV NLO applications. With UV absorption cut-off edges as short as 203 nm for (NH_4_)_2_Cd_2_Cl_3_F_3_ and 243 nm for (NH_4_)_2_Cd_2_Br_3_F_3_, these materials represent a significant advancement in the development of UV-transparent NLO materials. This study introduces a novel synthetic strategy for the design of d^10^ mixed halide systems with enhanced optical properties, offering promising candidates for future UV NLO technologies.

DOI: 10.1002/anie.2021XXXXX

Table of Contents

| **Section** | **Title** | **Page** |
| --- | --- | --- |
| **Experimental Procedures** | | S3 |
| Table S1. | Crystallographic Data for (NH_4_)_2_Cd_2_Cl_3_F_3_ and (NH_4_)_2_Cd_2_Br_3_F_3_. | S4 |
| Table S2. | Selected Bond Distances (Å) of (NH_4_)_2_Cd_2_Cl_3_F_3_. | S4 |
| Table S3. | Selected Bond Distances (Å) of (NH_4_)_2_Cd_2_Br_3_F_3_. | S4 |
| Table S4. | Selected Bond Angles (°) of (NH_4_)_2_Cd_2_Cl_3_F_3_. | S5 |
| Table S5. | Selected Bond Angles (°) of (NH_4_)_2_Cd_2_Br_3_F_3_. | S5 |
| Table S6. | Selected Bond Distances (Å) of NH_4_CdCl_3_. | S5 |
| Table S7. | Selected Bond Distances (Å) of NH_4_CdBr_3_. | S5 |
| Table S8. | Selected Bond Angles (°) of NH_4_CdCl_3_. | S6 |
| Table S9. | Selected Bond Angles (°) of NH_4_CdBr_3_. | S6 |
| Table S10. | Bond Valence Sum Calculations for (NH_4_)_2_Cd_2_Cl_3_F_3_ and (NH_4_)_2_Cd_2_Br_3_F_3_. | S6 |
| Table S11. | Hydrogen Bonds for (NH_4_)_2_Cd_2_Cl_3_F_3_ [Å and °]. | S7 |
| Table S12. | Hydrogen Bonds for (NH_4_)_2_Cd_2_Br_3_F_3_ [Å and °]. | S7 |
| Table S13. | Atomic Coordinates (Å) and Equivalent Isotropic Displacement Parameters (Å^2^) for (NH_4_)_2_Cd_2_Cl_3_F_3_. | S7 |
| Table S14. | Atomic Coordinates (Å) and Equivalent Isotropic Displacement Parameters (Å^2^) for (NH_4_)_2_Cd_2_Br_3_F_3_. | S7 |
| Table S15. | Anisotropic Displacement Parameters (Å^2^) for (NH_4_)_2_Cd_2_Cl_3_F_3_. | S8 |
| Table S16. | Anisotropic Displacement Parameters (Å^2^) for (NH_4_)_2_Cd_2_Br_3_F_3_. | S8 |
| Table S17. | Elemental Analysis Data of (NH_4_)_2_Cd_2_Cl_3_F_3_. | S8 |
| Table S18. | Elemental Analysis Data of (NH_4_)_2_Cd_2_Br_3_F_3_. | S8 |
| Table S19. | Local Dipole Moments of [CdCl_3_F_3_] in (NH_4_)_2_Cd_2_Cl_3_F_3_. | S9 |
| Table S20. | Local Dipole Moments of [CdBr_3_F_3_] in (NH_4_)_2_Cd_2_Br_3_F_3_. | S9 |
| Table S21. | NLO Properties and Band Gaps of d^10^ Metal Mixed Halide Compounds. | S10 |
| Figure S1. | Calculated and experimental PXRD patterns of (NH_4_)_2_Cd_2_Cl_3_F_3_. | S11 |
| Figure S2. | Calculated and experimental PXRD patterns of (NH_4_)_2_Cd_2_Br_3_F_3_. | S11 |
| Figure S3. | PXRD patterns of (NH_4_)_2_Cd_2_Cl_3_F_3_ after heating at 300 ℃. | S12 |
| Figure S4. | PXRD patterns of (NH_4_)_2_Cd_2_Br_3_F_3_ after heating at 700 ℃. | S12 |
| Figure S5. | PXRD patterns of (NH_4_)_2_Cd_2_Br_3_F_3_ after heating at 300 ℃. | S13 |
| Figure S6. | PXRD patterns of (NH_4_)_2_Cd_2_Br_3_F_3_ after heating at 700 ℃. | S13 |
| Figure S7. | (a) and (b) Crystals of (NH_4_)_2_Cd_2_Cl_3_F_3_ observed under cross-polarized light; (c) thickness of the selected crystal. | S14 |
| Figure S8. | (a) and (b) Crystals of (NH_4_)_2_Cd_2_Br_3_F_3_ observed under cross-polarized light; (c) thickness of the selected crystal. | S14 |
| Figure S9. | Calculated wavelength-dependent refractive index curves of (NH_4_)_2_Cd_2_Cl_3_F_3_ and (NH_4_)_2_Cd_2_Br_3_F_3_. | S15 |
| Figure S10. | TGA curves of (NH_4_)_2_Cd_2_Cl_3_F_3_ and (NH_4_)_2_Cd_2_Br_3_F_3_. | S15 |
| Figure S11. | IR spectrum of (NH_4_)_2_Cd_2_Cl_3_F_3_. | S16 |
| Figure S12. | IR spectrum of (NH_4_)_2_Cd_2_Br_3_F_3_. | S16 |
| Figure S13. | Band structures for (NH_4_)_2_Cd_2_Cl_3_F_3_. | S17 |
| Figure S14. | Band structures for (NH_4_)_2_Cd_2_Br_3_F_3_. | S17 |
| Figure S15. | Total and partial density of states for (a) (NH_4_)_2_Cd_2_Cl_3_F_3_ and (b) (NH_4_)_2_Cd_2_Br_3_F_3_. | S18 |
| Figure S16. | Block-shaped single crystal of (NH_4_)_2_Cd_2_Cl_3_F_3_. | S19 |
| Figure S17. | Block-shaped single crystal of (NH_4_)_2_Cd_2_Br_3_F_3_. | S19 |
| References |  | S20 |

Experimental Procedures

**Materials**

NH_4_F (96%, Thermo Scientific), NH_4_Cl (98%, Alfa Aesar), NH_4_Br (98%, Daejung), CdO (99.5%, Junsei), HCl (35−37%, Samchun), and HBr (47−49%, Daejung) were used without further purification.

**Synthesis**

Single crystals of the title compounds were grown via hydrothermal reactions. A mixture of CdO (5 mmol, 0.649 g) and NH_4_F (8 mmol, 0.259 g) was combined with 3 mL of deionized water and either 1.00 mL of HCl (for (NH_4_)_2_Cd_2_Cl_3_F_3_) or 1.25 mL of HBr (for (NH_4_)_2_Cd_2_Br_3_F_3_) in an 18 mL Teflon-lined autoclave. The mixture was sealed and heated to 180 °C over 3 h, maintained at that temperature for 10 h, and then cooled down over 20 h. The resulting clear solution was allowed to evaporate slowly at room temperature, yielding transparent block-shaped crystals over several days. The yields were 82% for (NH_4_)_2_Cd_2_Cl_3_F_3_ and 75% for (NH_4_)_2_Cd_2_Br_3_F_3_ based on CdO.

**Measurements**

Single crystal X-ray diffraction (SCXRD) analyses were performed at room temperature using a Bruker D8 Quest diffractometer equipped with a graphite-monochromated Mo Kα radiation source (λ = 0.71073 Å) at the Advanced Bio-interface Core Research Facility, Sogang University. After data integration using the SAINT program,^[26]^ absorption corrections were applied with the SADABS software.^[27]^ The structures were then solved using SHELXS-2013^[28]^ and further refined with SHELXL-2013, both run through the WinGX-2014 program.^[29]^

Powder X-ray diffraction (PXRD) analyses were conducted at room temperature using a Rigaku Miniflex 600 diffractometer equipped with a Cu Kα radiation source (λ = 1.54056 Å). The finely ground polycrystalline samples were analyzed over a 2*θ* range of 5–70° with a scanning speed of 20° min^-1^ and a scan step width of 0.02°.

Ultraviolet-visible (UV-vis) diffuse reflectance spectra were recorded using a PerkinElmer Lambda 1050 spectrophotometer, covering the range of 200–800 nm with a resolution of 1 nm at room temperature.

Infrared (IR) spectra were collected using a Thermo Scientific Nicolet iS50 FT-IR spectrometer equipped with an attenuated total reflection (ATR) accessory, covering the range of 800–4000 cm⁻¹ at room temperature. Thermogravimetric analysis (TGA) data were collected using a SCINCO TGA-N 1000 thermal analyzer. Polycrystalline samples were placed in alumina crucibles and heated from 30 to 900 ℃ at a heating rate of 10 ℃ min^-1^ under flowing air.

Birefringence (Δn) measurements for transparent crystals of both (NH_4_)_2_Cd_2_Cl_3_F_3_ and (NH_4_)_2_Cd_2_Br_3_F_3_ were performed using a polarized microscope (ZEISS Axiolab 5) equipped with a 546.1 nm monochromator.

**Calculations**

First-principles calculations for (NH₄)₂Cd₂Cl₃F₃ and (NH₄)₂Cd₂Br₃F₃ were conducted using the CASTEP software,^[30]^ a plane-wave pseudopotential total-energy package based on density functional theory (DFT)^[31]^ with norm-conserving pseudopotentials (NCP).^[32]^ The exchange-correlation energy was treated using the Perdew-Burke-Ernzerhof functional within the generalized gradient approximation.^[33]^ Core-electron interactions were modeled using on-the-fly generated NCP. The convergence criteria for the total energy and self-consistent field were set to 5 × 10^-6^ eV/atom and 1 × 10^-6^ eV/atom, respectively. For (NH₄)₂Cd₂Cl₃F₃, the valence electrons considered in the calculations included Cd 4d^10^5s^2^5p^0^, Cl 3s^2^3p^5^, F 2s^2^2p^5^, N 2s^2^2p^3^, and H 1s^1^ orbitals. Similarly, for (NH₄)₂Cd₂Br₃F₃, the valence electrons were Cd 4d^10^5s^2^5p^0^, Br 4s^2^4p^5^, F 2s^2^2p^5^, N 2s^2^2p^3^, and H 1s^1^ orbitals. The plane-wave basis sets were defined by a cutoff energy of 940 eV. The linear optical properties were investigated through the dielectric function Ɛ(ω) = Ɛ₁(ω) + iƐ₂(ω), where the imaginary part Ɛ₂(ω) was derived from the electronic structure, and the real part Ɛ₁(ω) was obtained via the Kramers-Kronig transformation. Based on these results, the refractive indices and birefringence (*Δn*) were subsequently calculated.

**Table S1.** Crystallographic Data for (NH_4_)_2_Cd_2_Cl_3_F_3_ and (NH_4_)_2_Cd_2_Br_3_F_3_.

|  | (NH_4_)_2_Cd_2_Cl_3_F_3_ | (NH_4_)_2_Cd_2_Br_3_F_3_ |
| --- | --- | --- |
| fw (g/mol) | 1272.70 | 1672.84 |
| space group | *R*32 | *R*32 |
| crystal system | Trigonal | Trigonal |
| *a = b* (Å) | 6.8915(2) | 7.00920(10) |
| *c* (Å) | 16.8503(3) | 17.3770(6) |
| *V* (Å^3^) | 693.05(4) | 739.34(3) |
| *Z* | 1 | 1 |
| *T* (K) | 298(2) | 298(2) |
| *λ* (Å) | 0.71073 | 0.71073 |
| *ρ* (g/cm^3^) | 3.049 | 3.757 |
| ^a^*R*(*F*) | 0.0186 | 0.0186 |
| ^b^*R_w_*(*F*_o_*^2^*) | 0.0471 | 0.0451 |
| *^a^R_p_* = Σ \|*I_o_*−*I_c_*\| / Σ *I_o_* and *^b^R_wp_* = [Σ*w* \|*I_o_*−*I_c_*\|^2^ / Σ*wI_o_*^2^]^1/2^ | | |

**Table S2.** Selected Bond Distances (Å) of (NH_4_)_2_Cd_2_Cl_3_F_3_.

| Bond | Distance (Å) |
| --- | --- |
| Cd(1)-F(1) × 3 | 2.294(3) |
| Cd(1)-Cl(1) × 3 | 2.5725(6) |

**Table S3.** Selected Bond Distances (Å) of (NH_4_)_2_Cd_2_Br_3_F_3_.

| Bond | Distance (Å) |
| --- | --- |
| Cd(1)-F(1) × 3 | 2.298(3) |
| Cd(1)-Br(1) × 3 | 2.6882(4) |

**Table S4.** Selected Bond Angles (°) of (NH_4_)_2_Cd_2_Cl_3_F_3_.

| Bond | Angle (°) |
| --- | --- |
| F(1)-Cd(1)-F(1) | 74.54(11) |
| F(1)-Cd(1)-Cl(1) | 90.63(4) |
| F(1)#1-Cd(1)-Cl(1) | 90.88(5) |
| F(1)#2-Cd(1)-Cl(1) | 161.37(7) |
| Cl(1)-Cd(1)-Cl(1) | 100.993(12) |

**Table S5.** Selected Bond Angles (°) of (NH_4_)_2_Cd_2_Br_3_F_3_.

| Bond | Angle (°) |
| --- | --- |
| F(1)-Cd(1)-F(1) | 75.53(13) |
| F(1)-Cd(1)-Br(1) | 92.03(5) |
| F(1)#1-Cd(1)-Br(1) | 90.60(6) |
| F(1)#2-Cd(1)-Br(1) | 163.20(9) |
| Br(1)-Cd(1)-Br(1) | 99.470(13) |

**Table S6.** Selected Bond Distances (Å) of NH_4_CdCl_3_.

| Bond | Distance (Å) |
| --- | --- |
| Cd(1)-Cl(1) | 2.660(3) |
| Cd(1)-Cl(1)#1 × 2 | 2.7142(15) |
| Cd(1)-Cl(2) × 2 | 2.6445(15) |
| Cd(1)-Cl(3) | 2.513(3) |

**Table S7.** Selected Bond Distances (Å) of NH_4_CdBr_3_.

| Bond | Distance (Å) |
| --- | --- |
| Cd(1)-Br(1) | 2.648(3) |
| Cd(1)-Br(2) × 2 | 2.7894(12) |
| Cd(1)-Br(3) | 2.796(3) |
| Cd(1)-Br(3)#1 × 2 | 2.8522(16) |

**Table S8.** Selected Bond Angles (°) of NH_4_CdCl_3_.

| Bond | Angle (°) |
| --- | --- |
| Cl(1)-Cd(1)-Cl(3) | 173.36(8) |
| Cl(2)-Cd(1)-Cl(3) | 172.56(8) |

**Table S9.** Selected Bond Angles (°) of NH_4_CdBr_3_.

| Bond | Angle (°) |
| --- | --- |
| Br(1)-Cd(1)-Br(3) | 177.28(7) |
| Br(2)-Cd(1)-Br(3) | 173.19(8) |

**Table S10.** Bond Valence Sum Calculations for (NH_4_)_2_Cd_2_Cl_3_F_3_ and (NH_4_)_2_Cd_2_Br_3_F_3_.

| (NH_4_)_2_Cd_2_Cl_3_F_3_ | | (NH_4_)_2_Cd_2_Br_3_F_3_ | |
| --- | --- | --- | --- |
| Cd1 | 2.04 | Cd1 | 2.04 |
| F1 | 0.56 | F1 | 0.56 |
| Cl1 | 0.80 | Cl1 | 0.80 |

**Table S11.** Hydrogen Bonds for (NH_4_)_2_Cd_2_Cl_3_F_3_ [Å and °].

| D-H...A | d(D-H) | d(H...A) | d(D...A) | ∠(DHA) |
| --- | --- | --- | --- | --- |
| N(1)-H(2)...Cl(1)#2 | 0.87(3) | 2.835(16) | 3.417(5) | 125.5(4) |
| N(1)-H(2)...Cl(1)#7 | 0.87(3) | 2.835(16) | 3.417(5) | 125.5(4) |
| N(1)-H(2)...Cl(1)#8 | 0.87(3) | 2.835(16) | 3.417(5) | 125.5(4) |
| N(1)-H(1)...F(1) | 0.87(3) | 1.87(4) | 2.726(3) | 171(14) |
| Symmetry operations: #1 -y+1,x-y,z #2 -x+y+1,-x+1,z #3 y+1/3,x-1/3,-z+2/3 #4 -y,x-y,z #5 -x+y,-x,z #6 y,x-1,-z+1 #7 x-1,y,z #8 -y,x-y-1,z | | | | |

**Table S12.** Hydrogen Bonds for (NH_4_)_2_Cd_2_Br_3_F_3_ [Å and °].

| D-H...A | d(D-H) | d(H...A) | d(D...A) | ∠(DHA) |
| --- | --- | --- | --- | --- |
| N(1)-H(2)...Br(1)#2 | 0.86(3) | 2.945(17) | 3.557(7) | 129.5(4) |
| N(1)-H(2)...Br(1)#7 | 0.86(3) | 2.945(17) | 3.557(7) | 129.5(4) |
| N(1)-H(2)...Br(1)#8 | 0.86(3) | 2.945(17) | 3.557(7) | 129.5(4) |
| N(1)-H(1)...F(1) | 0.86(3) | 1.91(3) | 2.766(3) | 171(10) |
| Symmetry operations: #1 -y+1,x-y,z #2 -x+y+1,-x+1,z #3 y+1/3,x-1/3,-z+2/3 #4 -y,x-y,z #5 -x+y,-x,z #6 y,x-1,-z+1 #7 x-1,y,z #8 -y,x-y-1,z | | | | |

**Table S13.** Atomic coordinates (Å) and equivalent isotropic displacement parameters (Å^2^) for (NH_4_)_2_Cd_2_Cl_3_F_3_.

|  | ***x*** | ***y*** | ***z*** | ***U(eq)*** |
| --- | --- | --- | --- | --- |
| Cd(1) | 0.6667 | 0.3333 | 0.4307(1) | 0.019(1) |
| Cl(1) | 0.6651(2) | 0 | 0.5 | 0.028(1) |
| F(1) | 0.4339(5) | 0.1005(5) | 0.3333 | 0.023(1) |
| N(1) | 0 | 0 | 0.3504(4) | 0.027(1) |

**Table S14.** Atomic Coordinates (Å) and Equivalent Isotropic Displacement Parameters (Å^2^) for (NH_4_)_2_Cd_2_Br_3_F_3_.

|  | ***x*** | ***y*** | ***z*** | ***U(eq)*** |
| --- | --- | --- | --- | --- |
| Cd(1) | 0.6667 | 0.3333 | 0.4268(1) | 0.019(1) |
| Br(1) | 0.6757(1) | 0 | 0.5 | 0.027(1) |
| F(1) | 0.4348(7) | 0.1015(7) | 0.3333 | 0.024(1) |
| N(1) | 0 | 0 | 0.3426(5) | 0.030(2) |

**Table S15.** Anisotropic Displacement Parameters (Å^2^) for (NH_4_)_2_Cd_2_Cl_3_F_3_.

|  | ***U^11^*** | ***U^22^*** | ***U^33^*** | ***U^23^*** | ***U^13^*** | ***U^12^*** |
| --- | --- | --- | --- | --- | --- | --- |
| Cd(1) | 0.020(1) | 0.020(1) | 0.016(1) | 0 | 0 | 0.010(1) |
| Cl(1) | 0.024(1) | 0.026(1) | 0.034(1) | 0.017(1) | 0.009(1) | 0.013(1) |
| F(1) | 0.022(1) | 0.022(1) | 0.023(2) | -0.001(1) | 0.001(1) | 0.009(1) |
| N(1) | 0.023(2) | 0.023(2) | 0.036(3) | 0 | 0 | 0.012(1) |

The anisotropic displacement factor exponent takes the form: *-*2*π^2^*[ *h^2^a*^2^U^11^ + ... +* 2 *h k a* b* U^12^* ]

**Table S16.** Anisotropic Displacement Parameters (Å^2^) for (NH_4_)_2_Cd_2_Br_3_F_3_.

|  | ***U^11^*** | ***U^22^*** | ***U^33^*** | ***U^23^*** | ***U^13^*** | ***U^12^*** |
| --- | --- | --- | --- | --- | --- | --- |
| Cd(1) | 0.019(1) | 0.019(1) | 0.017(1) | 0 | 0 | 0.010(1) |
| Br(1) | 0.023(1) | 0.026(1) | 0.033(1) | 0.014(1) | 0.007(1) | 0.013(1) |
| F(1) | 0.024(2) | 0.024(2) | 0.021(2) | -0.001(1) | 0.001(1) | 0.010(2) |
| N(1) | 0.023(2) | 0.023(2) | 0.043(5) | 0 | 0 | 0.012(1) |

The anisotropic displacement factor exponent takes the form: *-*2*π^2^*[ *h^2^a*^2^U^11^ + ... +* 2 *h k a* b* U^12^* ]

**Table S17.** Elemental Analysis Data of (NH_4_)_2_Cd_2_Cl_3_F_3_.

| Element | Calculated | Experimental |
| --- | --- | --- |
| N | 6.60 | 6.16 |
| H | 1.90 | 1.94 |
| Totals | 8.50 | 8.10 |

**Table S18.** Elemental Analysis Data of (NH_4_)_2_Cd_2_Br_3_F_3_.

| Element | Calculated | Experimental |
| --- | --- | --- |
| N | 5.02 | 4.47 |
| H | 1.45 | 1.49 |
| Totals | 6.47 | 5.96 |

**Table S19.** Local Dipole Moments of [CdCl_3_F_3_] in (NH_4_)_2_Cd_2_Cl_3_F_3_.

|  | ***x*** | ***y*** | ***z*** | DM (Debye) |
| --- | --- | --- | --- | --- |
| CdCl_3_F_3_ | 0.00 | 0.00 | 0.91 | 0.91 |
|  | 0.00 | 0.00 | 0.91 | 0.91 |
|  | 0.00 | 0.00 | -0.91 | 0.91 |
|  | 0.00 | 0.00 | -0.91 | 0.91 |
|  | 0.00 | 0.00 | 0.91 | 0.91 |
|  | 0.00 | 0.00 | -0.91 | 0.91 |

**Table S20.** Local Dipole Moments of [CdBr_3_F_3_] in (NH_4_)_2_Cd_2_Br_3_F_3_.

|  | ***x*** | ***y*** | ***z*** | DM (Debye) |
| --- | --- | --- | --- | --- |
| CdBr_3_F_3_ | 0.00 | 0.00 | 7.08 | 7.08 |
|  | 0.00 | 0.00 | 7.08 | 7.08 |
|  | 0.00 | 0.00 | -7.08 | 7.08 |
|  | 0.00 | 0.00 | -7.08 | 7.08 |
|  | 0.00 | 0.00 | 7.08 | 7.08 |
|  | 0.00 | 0.00 | -7.08 | 7.08 |

**Table S21.** NLO Properties and Band Gaps of d^10^ Metal Mixed Halide Compounds.

| Compounds | Space group | M-centered polyhedron | SHG | *E*_g_ (eV) |
| --- | --- | --- | --- | --- |
| β-HgBrCl | *P*2_1_2_1_2_1_ | [HgBrCl] | 2 $\times$ KDP | 3.40 |
| Hg_2_Br_3_I | *Cmc*2_1_ | [HgBrI] | 6.1 $\times$ KDP | 2.76 |
| Hg_2_BrI_3_ | *Cmc*2_1_ | [HgBrI] | 10.5 $\times$ KDP | 2.60 |
| Cs_2_Hg_2_Br_2_I_4_·H_2_O | *Pc* | [HgBrI]^2-^ | 6 $\times$ KDP | 2.82 |
| HgBrI | *Cmc*2_1_ | [HgBrI] | 12.2 $\times$ KDP | 2.70 |
| Rb_2_CdBrI_3_ | *Ama*2 | [CdBrI_3_]^2-^ | 2 $\times$ KDP | 4.01 |
| Rb_2_CdBr_2_I_2_ | *Ama*2 | [CdBr_2_I_2_]^2-^ | 4 $\times$ KDP | 3.35 |
| Cs_2_HgI_2_Cl_2_ | *P*2_1_ | [CdI_2_Cl_2_]^2-^ | 1 $\times$ KDP | 3.15 |
| (NH_4_)_2_Cd_2_Cl_3_F_3_ | *R*32 | [CdCl_3_F_3_]^4-^ | 3.0 $\times$ KDP | 5.80 |
| (NH_4_)_2_Cd_2_Br_3_F_3_ | *R*32 | [CdBr_3_F_3_]^4-^ | 8.0 $\times$ KDP | 4.74 |





**Figure S1.** Calculated and experimental PXRD patterns of (NH_4_)_2_Cd_2_Cl_3_F_3_.





**Figure S2.** Calculated and experimental PXRD patterns of (NH_4_)_2_Cd_2_Br_3_F_3_.





**Figure S3.** PXRD patterns of (NH_4_)_2_Cd_2_Cl_3_F_3_ after heating at 300 ℃.





**Figure S4.** PXRD patterns of (NH_4_)_2_Cd_2_Cl_3_F_3_ after heating at 700 ℃.





**Figure S5.** PXRD patterns of (NH_4_)_2_Cd_2_Br_3_F_3_ after heating at 300 °C.





**Figure S6.** PXRD patterns of (NH_4_)_2_Cd_2_Br_3_F_3_ after heating at 700 °C


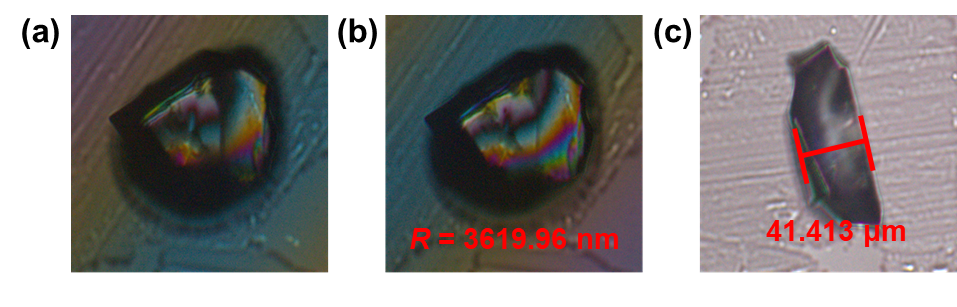


**Figure S7.** (a) and (b) Crystals of (NH₄)₂Cd₂Cl₃F₃ observed under cross-polarized light; (c) thickness of the selected crystal.


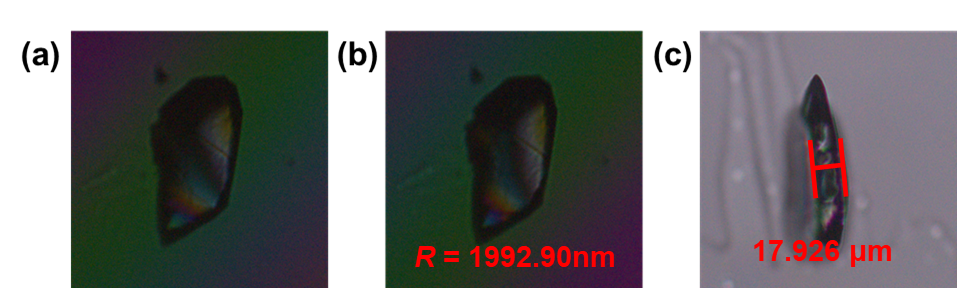


**Figure S8.** (a) and (b) Crystal of (NH_4_)_2_Cd_2_Br_3_F_3_ observed under cross-polarized light; (c) thickness of the selected crystal.

.
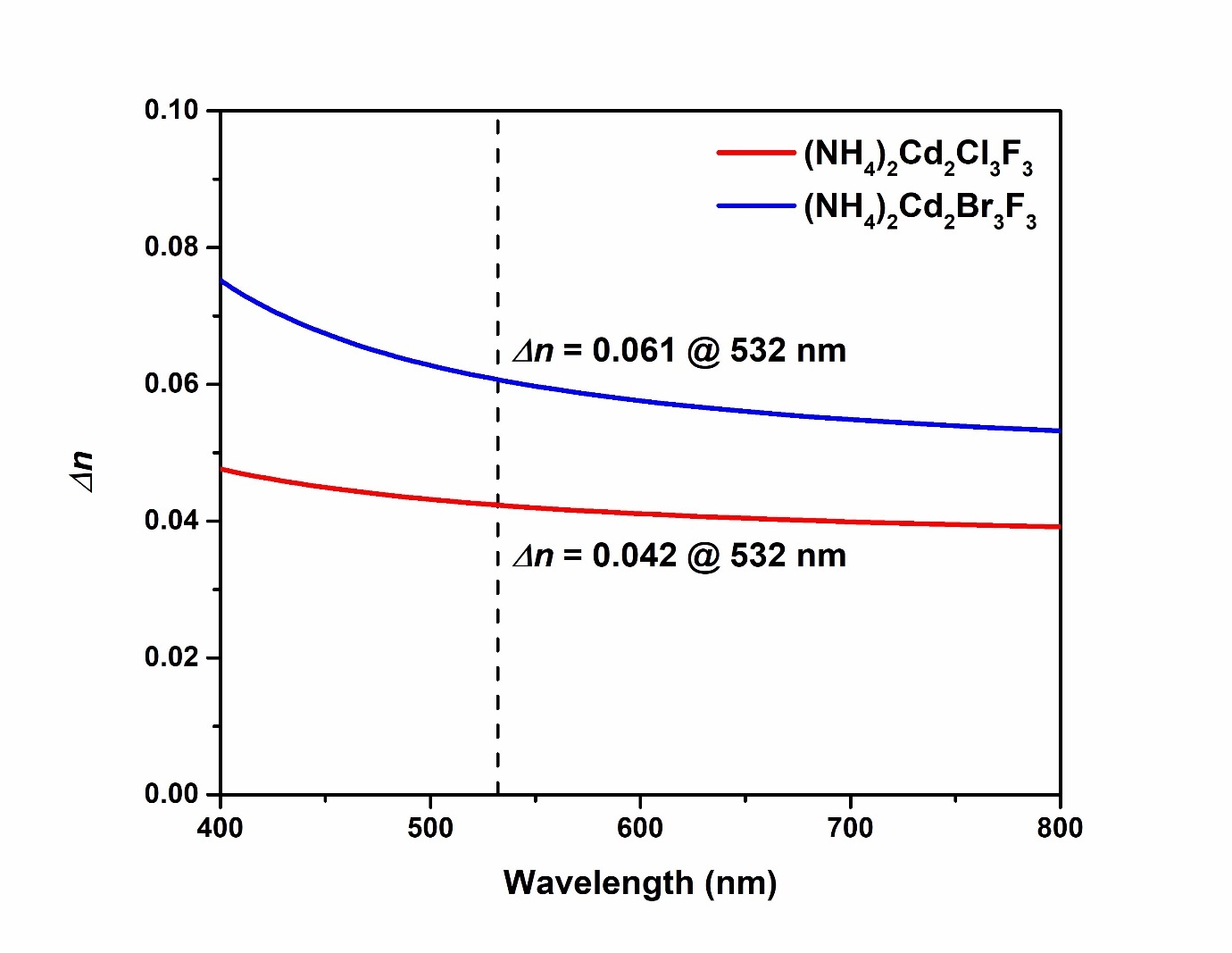


**Figure S9.** Calculated wavelength-dependent refractive index curves of (NH_4_)_2_Cd_2_Cl_3_F_3_ and (NH_4_)_2_Cd_2_Br_3_F_3_.

**

**

**Figure S10.** TGA curves of (NH_4_)_2_Cd_2_Cl_3_F_3_ and (NH_4_)_2_Cd_2_Br_3_F_3_.





**Figure S11.** IR spectrum of (NH_4_)_2_Cd_2_Cl_3_F_3_.





**Figure S12.** IR spectrum of (NH_4_)_2_Cd_2_Br_3_F_3_.


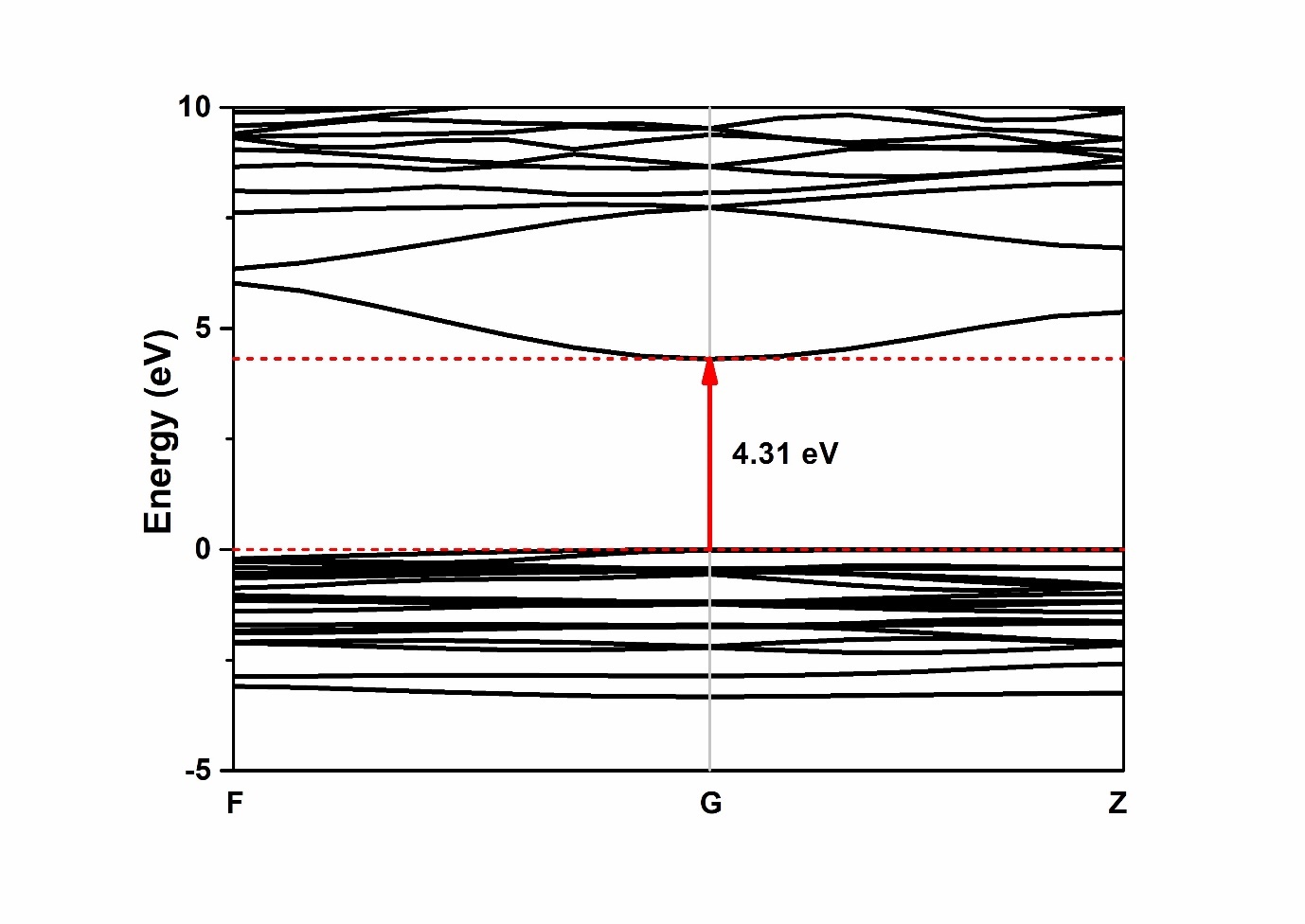


**Figure S13.** Band structures for (NH₄)₂Cd₂Cl₃F_3_.
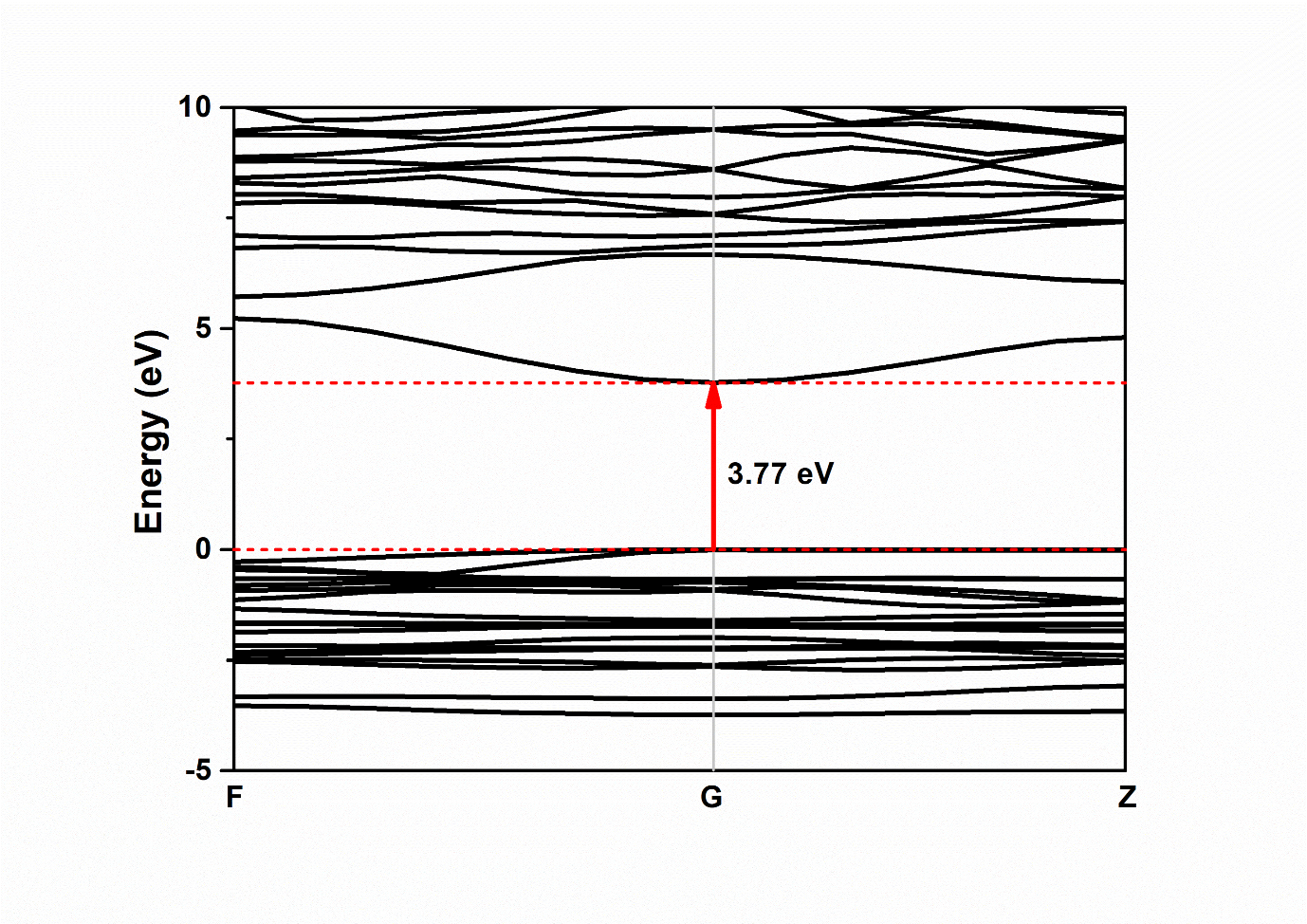


**Figure S14.** Band structures for (NH₄)₂Cd₂Br₃F_3_.


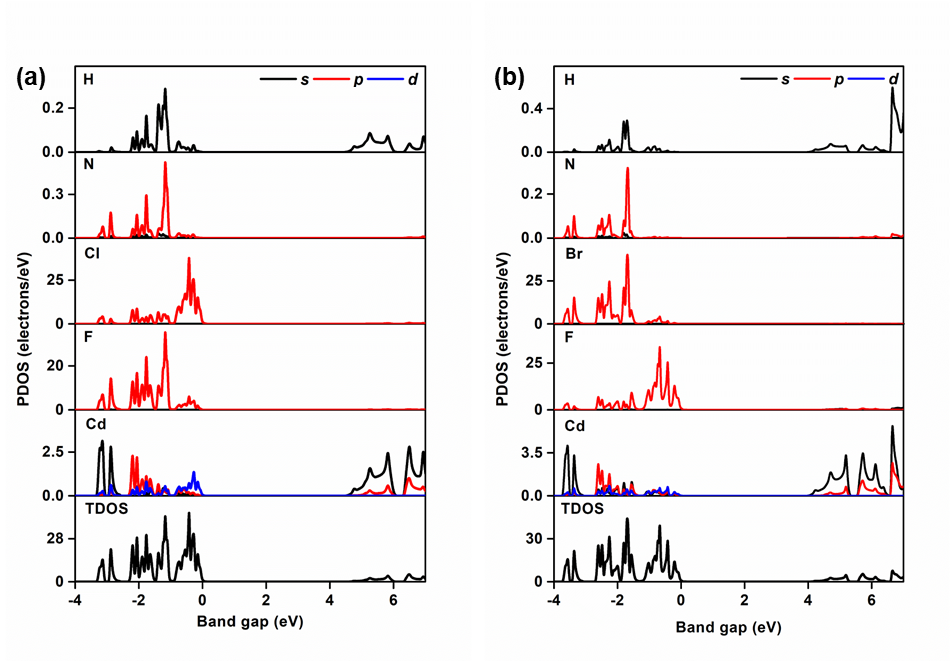


**Figure S15.** Total and partial density of states for (a) (NH₄)₂Cd₂Cl₃F_3_ and (b) (NH₄)₂Cd₂Br₃F_3_.

#
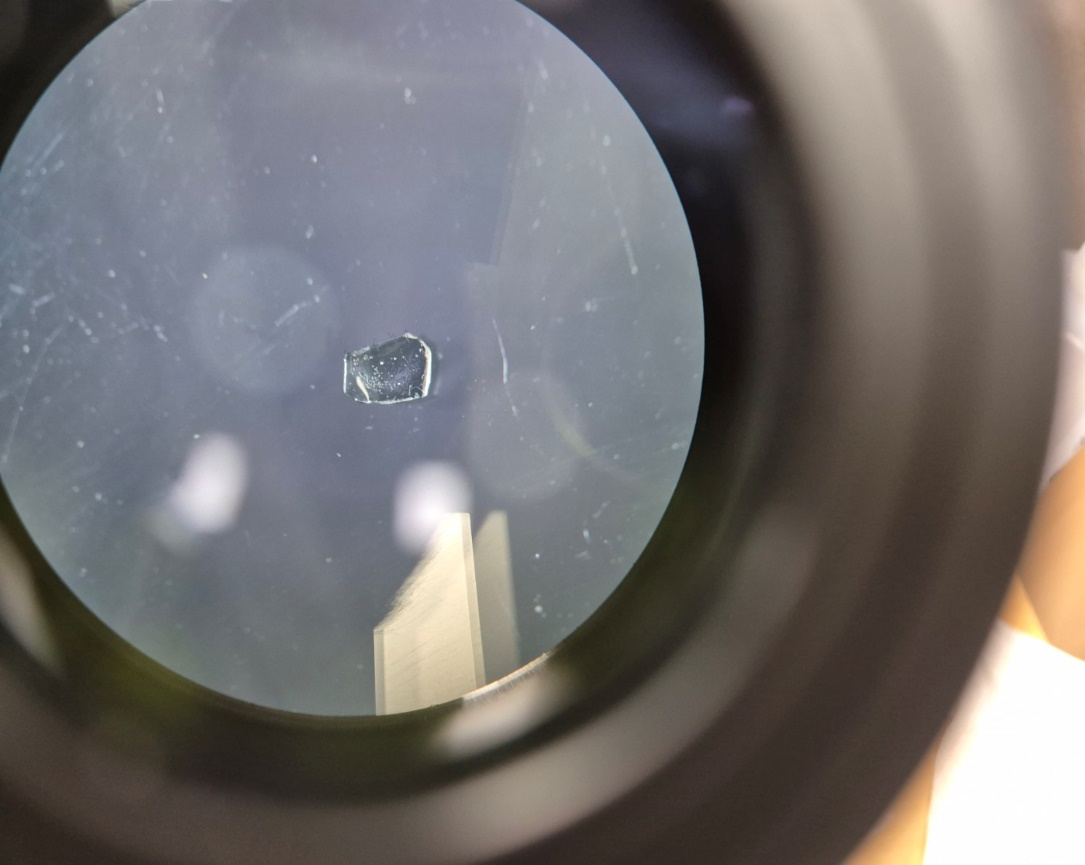


**Figure S16.** Block-shaped single crystal of (NH_4_)_2_Cd_2_Cl_3_F_3_ (maximum width: 5mm).


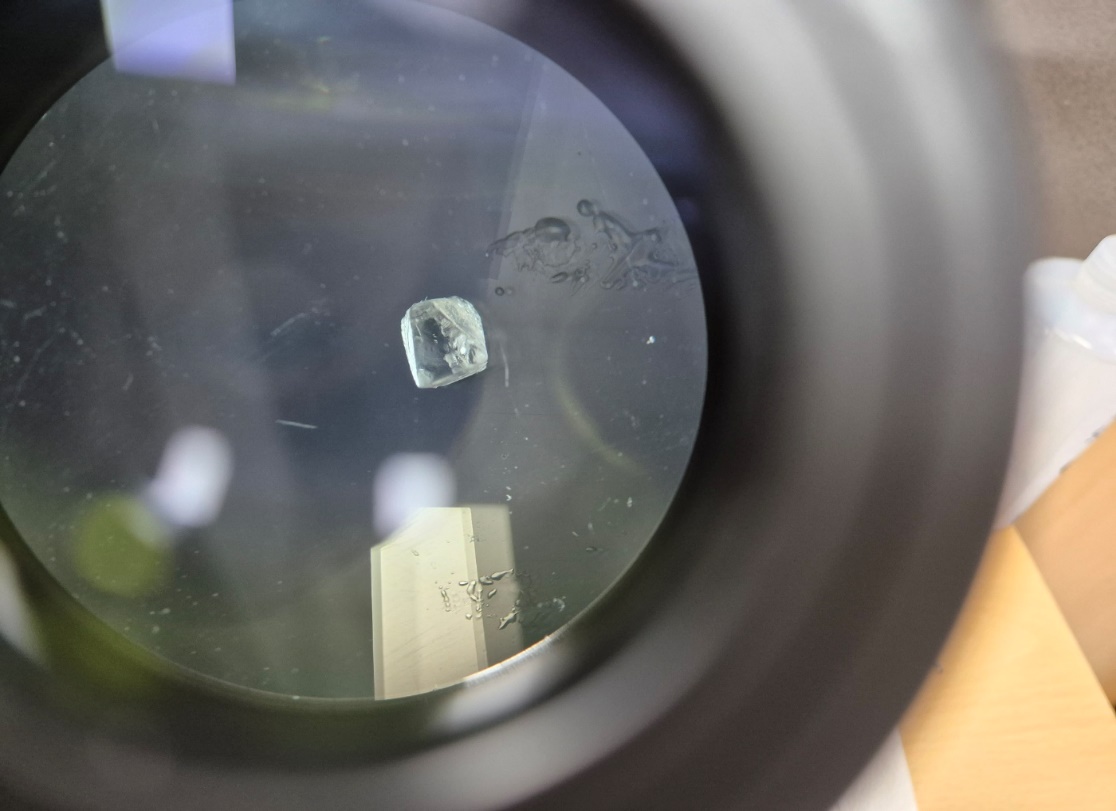


**Figure S17.** Block-shaped single crystal of (NH_4_)_2_Cd_2_Br_3_F_3_ (maximum width: 5mm).

# References

[26] SAINT, Version 7.60 A, Bruker Analytical X-ray Instruments, Inc.*,* Madison, WI **2008**.

[27] G. M. Sheldrick, *Acta Crystallogr. C* **2015**, *71*, 3.

[28] R. H. Blessing, *Acta Crystallogr. Sect. A: Found. Crystallogr.* **1995**, *51*, 33-38.

[29] L. J. Farrugia, *J. Appl. Crystallogr.* **2012**, *45*, 849-854.

[30] S. J. Clark, M. D. Segall, C. J. Pickard, P. J. Hasnip, M. I. Probert, K. Refson, M. C. Payne, *Z. Kristallogr.-Cryst. Mater.* **2005**, *220*, 567-570.

[31] J. Lin, A. Qteish, M. Payne, V. Heine, *Phys. Rev. B* **1993**, *47*, 4174.

[32] A. M. Rappe, K. M. Rabe, E. Kaxiras, J. Joannopoulos, *Phys. Rev. B* **1990**, *41*, 1227.

[33] J. P. Perdew, K. Burke, M. Ernzerhof, *Phys. Rev. Lett.* **1996**, *77*, 3865.

# Author Contributions

Seunghun Choi: Conceptualization, investigation, visualization, writing – original draft

Yang Li: Investigation, data curation

Yunseung Kuk: Investigation, data curation

Kang Min Ok: Conceptualization, funding acquisition, project administration, writing – review & editing. lead
